# Nutrient intakes in an Italian population of infants during the complementary feeding period

**DOI:** 10.1017/S136898001800201X

**Published:** 2018-08-30

**Authors:** Federica Concina, Paola Pani, Giulia Bravo, Fabio Barbone, Claudia V Carletti, Alessandra Knowles, Luca Ronfani, Maria Parpinel

**Affiliations:** 1 Clinical Epidemiology and Public Health Research Unit, Institute for Maternal and Child Health – IRCCS ‘Burlo Garofolo’, Via dell’Istria 65/1, 34137 Trieste, Italy; 2 Department of Medicine, University of Udine, Udine, Italy; 3 Scientific Direction, Institute for Maternal and Child Health – IRCCS ‘Burlo Garofolo’, Trieste, Italy

**Keywords:** Prospective cohort study, Infants, Complementary feeding, Energy and nutrient intakes, Italian Dietary Reference Values

## Abstract

**Objective:**

To describe the nutrient intakes of an Italian cohort of infants at 6, 9 and 12 months of age.

**Design:**

Dietary data were collected using a food diary at three follow-ups (6, 9 and 12 months of age of infants). The infants’ dietary data were used to estimate nutrient intakes using the Italian food composition database integrated with data from nutritional labels and the literature. The mean and standard deviation, median and interquartile range, minimum and maximum, and 5th, 25th, 75th and 95th percentiles were calculated for the daily intake of twenty-eight nutrients, with sex differences evaluated using parametric/non-parametric statistical methods.

**Setting:**

A prospective population-based birth cohort.

**Subject:**

Infants (*n* 400) living in the urban area of Trieste (Italy).

**Results:**

The sex distribution was fairly balanced at each follow-up. The mean daily intakes of energy and the other twenty-seven nutrients considered were greater in males at all follow-ups. In particular, a significant statistical difference was observed in higher male consumption of cholesterol at 9 months and in energy and carbohydrate intakes at 12 months (*P* < 0·05). The mean daily intake of proteins was greater than that recommended by the Italian Dietary Reference Values at all follow-ups.

**Conclusions:**

These preliminary results provide a useful basis for understanding the nutrient intake patterns of infants in this area of Italy during the first year of life.

Understanding the dietary habits and the nutritional intake of infants during the first 2 years of life is important because this period, which covers breast-feeding and complementary feeding, is characterized by high nutrient requirements and critical dietary changes that have an impact on the development of food preferences and on short- and long-term health^(^ ^1^ ^,^ ^2^ ^)^.

Inadequate complementary feeding, as well as maternal malnutrition and inappropriate breast-feeding, can have direct and indirect negative consequences on child health, such as inadequate growth velocity, infections, obesity, CVD, autoimmune diseases (coeliac disease and type 1 diabetes) and atopic disorders^(^ ^3^ ^)^.

In 2003, the WHO and UNICEF published a ‘Global Strategy for Infant and Young Child Feeding’ emphasizing that inadequate feeding practices are a major risk factor for morbidity and mortality in the first part of infancy^(^ ^4^ ^)^. Subsequently, in 2017, the European Society of Pediatric Gastroenterology, Hepatology and Nutrition published a position paper on complementary feeding (latest edition), reviewing differences in knowledge and practices among countries and summarizing the limited available scientific evidence on the short- and long-term health effects of timing and composition of complementary foods^(^ ^5^ ^)^.

Unfortunately, there are very few studies describing nutritional practices at different ages during the first 2 years of life and determining nutrient intakes in detail. Among international studies, the most relevant are four large cohort studies: the Norwegian Mother and Child Cohort Study (MoBa)^(^ ^6^ ^)^, the Dortmund Nutritional and Anthropometric Longitudinally Designed Study (DONALD)^(^ ^7^ ^)^, the Avon Longitudinal Study of Pregnancy and Childhood (ALSPAC)^(^ ^8^ ^)^ and the ‘Public health impact of long-term, low-level mixed element exposure in susceptible population strata’ study (PHIME)^(^ ^9^ ^)^. Although these studies all involve children and parents, their aims are substantially different. The MoBa study examines the causes of serious diseases by estimation of specific exposure–outcome associations among children and parents^(^ ^6^ ^)^. The DONALD study focuses on the relationship between diet, nutrition and development of children and adolescents during their growth period^(^ ^7^ ^)^. The ALSPAC study is designed to determine how the genotype combines with environmental pressures to influence health and development of children^(^ ^8^ ^)^. Finally, the PHIME study aims at improving the integrated health-risk assessment of long-term, low-level environmental exposure to toxic and essential metals via food^(^ ^9^ ^)^. In Italy, PHIME was designed to investigate the effects of prenatal low-level Hg exposure from fish and seafood consumption on the neuro- and physiological development of infants in the first 18 months of life^(^ ^9^ ^)^. During the same period, a Trieste Infants Food cohort (TIF cohort), partially overlapping with the Italian component of the PHIME study, was set up to study nutrition in the first 3 years of life in the north-east of Italy^(^ ^10^ ^)^.

The main objective of the present paper is to provide a descriptive analysis of the intakes of nutrients by infants aged 6, 9 and 12 months, as part of the TIF cohort, according to sex^(^ ^10^ ^)^. The secondary objective is to compare the results with national and international recommendations^(^ ^11^ ^,^ ^12^ ^)^.

To our knowledge, the present study is the first to provide a detailed description of the dietary intake of an Italian infant population at different ages during the second semester of life, when complementary feeding is established.

## Methods

A total of 400 infants born at the Institute of Maternal and Child Health of Trieste, Italy, between 2006 and 2007 were enrolled at birth. The Ethics Committee of the Institute of Maternal and Child Health – IRCCS ‘Burlo Garofolo’ of Trieste approved the protocol of the study and all participating subjects were informed and consented to participate. The study design, protocol and sampling procedures are described elsewhere^(^ ^10^ ^)^.

Dietary data were collected using a 3d dietary record (food diary) at 6, 9 and 12 months of age. The food diary was given to mothers on the occasion of the first contact, together with instructions on how to record type, quantity and method of feeding over a 24h period on three separate non-consecutive days, including one at the weekend. The instructions included a table with a list of household implements (e.g. teaspoons) that could be used at home to weigh foods and fluids, with an estimate of the equivalent in grams. A telephone contact number was provided to mothers in case they had questions regarding the completion of the food diary.

Data extracted from the food diaries were analysed using the Microdiet software version 2.8.6 (Downlee Systems Ltd, High Peak, UK) containing the Italian food composition database for epidemiological studies^(^ ^13^ ^)^, integrated with information from nutritional labels (e.g. baby food) and, in the case of human milk, from the literature^(^ ^14^ ^,^ ^15^ ^)^. For human milk, the duration of each feed in combination with the frequency of breast-feeding was used to estimate the volume of milk, as suggested by Cameron and Hofvander^(^ ^16^ ^)^. The complete methodology for coding and conversion of food intakes into nutrient intakes is described in Concina *et al*.^(^ ^17^ ^)^. Additional demographic, education, social and anthropometric data on the mother, the father and the infant were obtained from a questionnaire^(^ ^9^ ^)^. All procedures were conducted by trained food technologists and nutritionists, who were fully familiar with brand names, composition of commercial products, food preparation methods and in the management of food composition data.

Twenty-eight food components, defined by the Italian food composition database research group^(^ ^13^ ^)^, were considered for nutritional analysis: total proteins, carbohydrates (available, soluble, starch, fibre), lipids (total, saturated, monounsaturated and polyunsaturated fatty acids; oleic, linoleic and linolenic acids; cholesterol), minerals (Na, K, Ca, Fe, Zn) and vitamins (thiamin, riboflavin, vitamin B_6_, vitamin C, vitamin D, vitamin E expressed as α-tocopherol equivalents, retinol, retinol equivalents, niacin, folic acid).

### Statistical analysis

Categorical data are presented as number and percentage; continuous data as mean and standard deviation. For each infant, the mean daily intakes of energy, macronutrients and micronutrients were calculated on a 3d observation basis, excluding the use of supplements.

The mean and standard deviation, median and interquartile range (IQR), minimum and maximum, and 5th, 25th, 75th and 95th percentiles were calculated for daily intake of each nutrient in all infants and by sex. The percentage contribution of each macronutrient to energy intake was estimated for all infants. The nutrient intakes were then compared with the Italian Dietary Reference Values (DRV)^(^ ^11^ ^)^ and the WHO feeding and nutrition recommendations for infants and young children^(^ ^12^ ^)^, when available. More precisely, the DRV were expressed using different indices such as the Suggested Dietary Target, Adequate Intake, Reference Intake range for macronutrients, Average Requirement and Population Reference Intake, while WHO recommendations were expressed using the Recommended Daily Intake.

To detect significant sex differences for each nutrient and in terms of energy intake, a comparative analysis was conducted at each follow-up using *t* tests or the corresponding non-parametric test (Wilcoxon, Mann–Whitney) if a non-normal distribution of data was seen by the Kolmogorov–Smirnof test. Statistical significance for all tests was set at a *P* value of 0·05.

Analyses were conducted using the statistical software package SAS version 9.4 for Windows.

## Results

The characteristics of mothers and infants at enrolment and delivery, respectively, are reported in detail in Table 1. The differences in sample size reported in Table 1 for some variables are due to missing data in the questionnaires.

**Table 1:** Characteristics of mothers and infants at enrolment and delivery in the Trieste Infants Food cohort (*n* 400)

Characteristic	*n*	%
Nationality of mother		
Italian	351	87·8
Foreign	49	12·3
Not reported	0	0·0
Marital status		
Married/living with partner	318	80·0
Separated/divorced	13	3·3
Single/not living with partner	18	5·0
Not reported	51	12·3
Maternal education level		
None	1	0·3
Completed primary school	6	2·0
Completed secondary school	52	13·0
Completed high school or equivalent	154	39·0
Bachelor degree or higher	135	33·8
Not reported	52	13·0
Maternal occupational status		
Yes	306	77·0
No	17	4·3
Not reported	77	19·3
Sex of child		
Male	173	43·3
Female	172	42·0
Not reported	55	13·8
	Mean	sd
Maternal age (years; *n* 348)	33·0	4·7
Birth weight of child (g; *n* 344)	3394·6	456·0
Birth length of child (cm; *n* 342)	50·4	2·1

The mean maternal age was 33·0 (sd 4·7) years. Most women were Italian (87·8%), married (80·0%), in employment (77·0%) and had a medium–high level of education (72·8%).

**Fig. 1:**
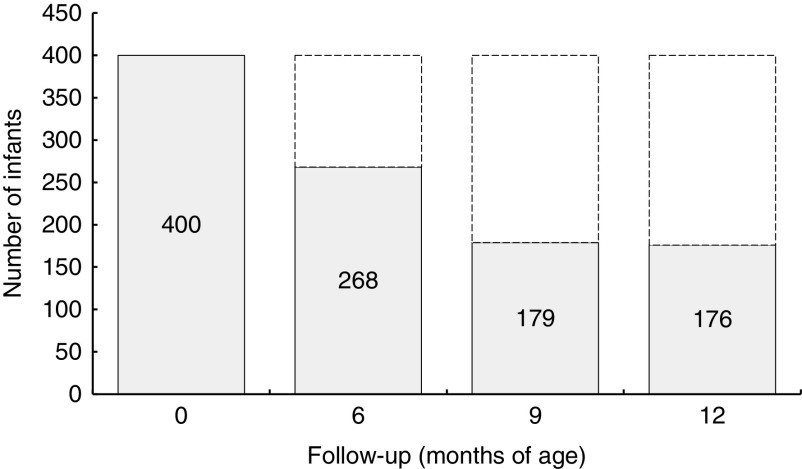
Distribution of infants at each follow-up: the Trieste Infants Food cohort

Infants were equally distributed by sex: 173 males and 172 females; for fifty-five infants, gender information was not reported. The mean birth weight and length of infants at delivery was 3394·6 (sd 456·0) g and 50·4 (sd 2·1) cm, respectively. The number food diaries available for analysis were 268 at 6 months, 179 at 9 months and 176 at 12 months of age of the infants (Fig. 1).

A dropout analysis of data at 6, 9 and 12 months showed that the sample was statistically representative in terms of maternal age at enrolment (*P*=0·03 at 6 months and *P*=0·02 at 12 months), maternal age at delivery (*P*=0·03 at 6 and 9 months and *P*=0·01 at 12 months), education (*P*=0·00 at 6 months, *P*=0·04 at 9 months and *P*=0·02 at 12 months) and occupation (*P*=0·03 at 6 months; data not shown).

The rates of breast-feeding, alone or in combination with infant formula or cow’s milk, or both, were 70·0% at 6 months, 54·8% at 9 months and 39·7% at 12 months. Moreover, at 6 months only 6·0% of infants were breast-fed exclusively while at the other follow-ups, none were breast-fed or formula-fed exclusively (data not shown).

Daily energy, macronutrient and micronutrient intakes for all infants, divided by sex, are shown in Tables 2, 3 and 4 (for infants at 6, 9 and 12 months of age, respectively).

**Table 2:** Daily energy, macronutrient and micronutrient intakes of Italian infants at 6 months of age in the Trieste Infants Food cohort

	All infants (*n* 268)				Females (*n* 132)				Males (*n* 136)			
Nutrient	Mean	sd	Median	IQR	Mean	sd	Median	IQR	Mean	sd	Median	IQR
Energy (kJ)	2437·7	803·3	2312·7	887·3	2360·0	706·8	2250·7	773·9	2513·1	883·1	2344·2	1180·5
Total proteins (g)	15·6	9·6	14·1	9·2	15·3	7·8	14·2	7·8	15·9	11·1	14·1	10·5
Total lipids (g)	25·5	8·2	23·8	9·5	25·0	7·9	23·3	9·1	26·0	8·4	24·3	9·5
SFA (g)	9·5	2·9	8·9	3·4	9·3	2·8	8·8	3·1	9·6	2·9	9·0	3·8
MUFA (g)	9·9	4·0	9·1	4·5	9·6	3·7	9·0	4·7	10·1	4·2	9·2	4·1
Oleic acid (g)	9·5	3·9	8·7	4·0	9·2	3·7	8·5	4·3	9·8	4·0	9·0	3·8
PUFA (g)	3·3	1·6	2·9	1·7	3·3	1·6	2·8	2·1	3·3	1·5	3·0	1·6
Linoleic acid (g)	2·8	1·3	2·4	1·4	2·8	1·4	2·4	1·7	2·7	1·3	2·4	1·3
Linolenic acid (g)	0·9	0·5	0·8	0·5	0·9	0·5	0·8	0·6	0·9	0·4	0·8	0·4
Available carbohydrates (g)	77·6	28·9	71·4	33·7	74·1	25·8	70·1	27·4	80·9	31·3	74·0	42·3
Soluble carbohydrates (g)	47·9	13·2	46·7	16·9	46·0	12·0	44·8	15·4	49·7	14·2	47·5	17·9
Starch (g)	12·7	13·4	9·3	18·1	12·5	13·0	9·0	18·2	13·0	13·8	9·9	16·9
Fibre (g)	4·2	3·9	3·3	4·8	3·8	3·4	3·1	4·4	4·6	4·4	3·6	5·0
Cholesterol (mg)	62·8	43·7	66·1	72·4	59·5	43·5	65·1	75·0	66·1	43·7	67·5	63·2
Na (mg)	373·1	321·5	253·4	317·5	350·1	271·9	251·3	272·5	395·3	362·9	257·1	374·5
K (mg)	747·4	408·3	635·7	399·1	716·7	379·5	617·4	356·7	777·2	433·8	650·7	444·4
Ca (mg)	383·5	184·0	346·4	245·0	391·6	189·5	349·2	234·8	375·6	178·8	316·3	244·6
Fe (mg)	3·6	4·2	2·5	5·0	3·8	5·3	2·7	4·8	3·4	3·0	2·3	5·1
Zn (mg)	3·5	1·3	3·3	1·5	3·5	1·3	3·3	1·6	3·5	1·3	3·3	1·6
Thiamin (mg)	0·5	0·4	0·4	0·5	0·5	0·5	0·4	0·5	0·5	0·3	0·4	0·5
Riboflavin (mg)	0·7	0·5	0·5	0·6	0·7	0·6	0·6	0·6	0·6	0·4	0·5	0·6
Vitamin B_6_ (mg)	0·5	0·5	0·5	0·5	0·5	0·6	0·4	0·4	0·5	0·4	0·5	0·5
Vitamin C (mg)	62·9	32·4	56·1	44·3	61·8	32·0	53·5	45·5	63·9	32·9	56·7	43·5
Vitamin D (µg)	2·7	3·3	0·3	5·1	2·9	3·4	0·6	5·4	2·5	3·2	0·3	5·0
Vitamin E, α-TE (mg)	1·4	1·4	1·0	1·6	1·2	1·3	0·8	1·3	1·5	1·5	1·2	2·1
Retinol (µg)	94·8	142·2	16·6	150·8	99·9	144·5	19·3	179·9	89·8	140·2	10·9	120·4
RE (µg)	279·8	366·5	144·0	393·1	253·1	347·1	133·4	315·7	305·8	383·9	165·7	469·7
Niacin (mg)	4·4	5·2	3·4	3·9	4·7	6·7	3·4	3·7	4·2	3·1	3·4	4·1
Folate (µg)	97·7	48·0	87·7	51·0	95·3	45·1	88·6	49·9	99·9	50·8	86·2	56·5

IQR, interquartile range; α-TE, α-tocopherol equivalents; RE, retinol equivalents. No significant difference by sex was detected.

**Table 3:** Daily energy, macronutrient and micronutrient intakes of Italian infants at 9 months of age in the Trieste Infants Food cohort

	All infants (*n* 179)				Females (*n* 89)				Males (*n* 90)			
Nutrient	Mean	sd	Median	IQR	Mean	sd	Median	IQR	Mean	sd	Median	IQR
Energy (kJ)	3213·2	890·2	3220·9	1000·5	3208·2	962·1	3167·6	1179·4	3218·2	818·4	3242·0	900·6
Total proteins (g)	27·9	11·4	26·7	13·1	27·3	10·3	26·7	13·4	28·5	12·5	26·4	13·1
Total lipids (g)	30·6	9·8	29·8	13·2	31·1	10·5	30·4	14·1	30·0	9·0	28·3	11·6
SFA (g)	10·6	3·7	10·3	4·2	10·6	3·8	10·5	4·6	10·6	3·6	10·0	3·7
MUFA (g)	11·5	4·5	10·6	5·6	11·5	4·6	10·6	5·6	11·4	4·4	10·6	5·2
Oleic acid (g)	10·6	4·6	10·1	5·6	10·7	4·7	10·1	6·0	10·6	4·4	10·0	4·3
PUFA (g)	3·5	1·6	3·2	1·9	3·6	1·8	3·2	2·0	3·4	1·4	3·1	1·9
Linoleic acid (g)	2·9	1·4	2·5	1·6	3·0	1·6	2·7	1·6	2·7	1·2	2·5	1·6
Linolenic acid (g)	0·5	0·2	0·4	0·3	0·5	0·2	0·4	0·3	0·5	0·2	0·4	0·3
Available carbohydrates (g)	101·7	30·5	98·6	36·2	100·6	33·3	98·6	40·6	102·7	27·6	98·8	31·0
Soluble carbohydrates (g)	43·4	15·4	43·4	19·0	41·2	15·9	40·4	19·8	45·5	14·6	45·3	16·1
Starch (g)	29·5	19·2	25·0	24·6	29·2	18·7	27·2	25·7	29·8	19·7	24·9	24·7
Fibre (g)	7·0	3·6	6·6	4·6	6·8	3·6	6·2	3·9	7·3	3·5	6·8	4·8
Cholesterol (mg)	69·3	42·0	64·1	57·2	62·6*	39·1	60·9	50·3	75·8*	43·9	70·0	47·5
Na (mg)	671·2	492·1	521·9	499·5	633·5	499·1	498·4	419·9	708·6	484·8	542·1	619·7
K (mg)	1080·5	505·5	1031·6	618·3	1039·4	448·0	1008·2	596·8	1121·1	556·1	1044·4	657·3
Ca (mg)	536·1	221·6	523·9	313·3	543·6	220·8	532·7	327·8	528·7	223·4	508·4	301·5
Fe (mg)	6·3	14·7	5·0	4·4	7·5	20·7	4·9	4·3	5·2	2·7	5·0	4·4
Zn (mg)	3·6	1·8	3·5	2·5	3·7	1·7	3·7	2·7	3·4	1·8	3·4	2·3
Thiamin (mg)	0·6	0·3	0·6	0·4	0·6	0·3	0·5	0·5	0·6	0·3	0·6	0·3
Riboflavin (mg)	0·8	0·4	0·8	0·5	0·8	0·4	0·8	0·5	0·8	0·4	0·7	0·5
Vitamin B_6_ (mg)	0·8	0·4	0·8	0·4	0·8	0·4	0·8	0·4	0·8	0·4	0·8	0·4
Vitamin C (mg)	70·5	34·9	62·8	48·8	69·2	33·2	61·8	38·0	71·7	36·7	64·4	53·3
Vitamin D (µg)	2·3	2·5	0·7	4·1	2·6	2·7	1·9	4·4	1·9	2·4	0·5	3·2
Vitamin E, α-TE (mg)	2·8	1·6	2·4	2·2	2·6	1·6	2·3	2·3	2·9	1·7	2·5	1·8
Retinol (µg)	72·5	68·8	50·7	69·4	69·3	62·3	45·2	64·8	75·6	74·8	59·9	72·1
RE (µg)	515·0	394·4	423·7	503·6	506·5	398·3	384·6	513·4	523·5	392·6	453·1	461·9
Niacin (mg)	6·4	3·4	6·0	4·3	6·5	3·5	6·0	4·8	6·4	3·3	6·3	4·0
Folate (µg)	122·7	61·3	107·2	65·3	122·3	56·5	109·5	60·3	123·2	66·0	102·6	65·7

IQR, interquartile range; α-TE, α-tocopherol equivalents; RE, retinol equivalents. Mean value was significantly different between sexes: *P<0·05.

**Table 4:** Daily energy, macronutrient and micronutrient intakes of Italian infants at 12 months of age in the Trieste Infants Food cohort

	All infants (*n* 176)				Females (*n* 85)				Males (*n* 91)			
Nutrient	Mean	sd	Median	IQR	Mean	sd	Median	IQR	Mean	sd	Median	IQR
Energy (kJ)	3357·3	907·1	3274·3	1027·3	3242·8*	907·6	3138·3	1147·6	3464·4*	898·5	3509·1	948·2
Total proteins (g)	32·5	11·2	32·1	13·4	32·6	11·4	32·3	12·7	32·5	11·1	32·0	14·3
Total lipids (g)	30·2	10·0	29·2	11·6	29·6	9·1	28·7	11·2	30·7	10·7	30·1	12·1
SFA (g)	12·1	4·6	11·1	6·3	12·0	4·3	11·1	5·8	12·1	4·9	11·1	6·7
MUFA (g)	11·2	4·7	10·6	5·3	10·8	4·0	10·0	4·9	11·6	5·3	11·4	6·6
Oleic acid (g)	10·2	4·5	9·8	4·7	9·9	3·9	9·3	4·7	10·6	5·0	10·3	4·7
PUFA (g)	3·0	1·4	2·6	1·7	2·9	1·3	2·5	1·5	3·1	1·5	2·8	1·7
Linoleic acid (g)	2·4	1·3	2·0	1·5	2·3	1·2	1·9	1·5	2·5	1·3	2·2	1·5
Linolenic acid (g)	0·5	0·2	0·4	0·3	0·5	0·2	0·4	0·3	0·5	0·2	0·4	0·3
Available carbohydrates (g)	106·8	30·8	104·0	44·8	100·8*	31·2	93·8	38·4	112·4*	29·4	113·0	45·8
Soluble carbohydrates (g)	46·1	15·7	45·0	17·0	43·1*	13·4	42·3	13·2	48·9*	17·2	48·3	22·8
Starch (g)	36·7	21·7	33·8	30·8	33·6*	21·5	29·6	25·5	39·7*	21·6	39·7	33·9
Fibre (g)	7·0	3·2	7·0	4·3	6·5	2·9	6·0	4·0	7·5	3·5	7·2	4·1
Cholesterol (mg)	100·6	59·0	88·0	56·5	100·8	6·0	87·9	57·9	100·5	60·3	88·1	56·1
Na (mg)	721·6	397·7	654·1	467·5	655·8	334·8	575·9	404·1	783·1	441·7	710·6	679·5
K (mg)	1290·1	510·2	1227·3	703·4	1291·9	492·0	1265·2	640·8	1288·3	529·3	1190·5	754·5
Ca (mg)	595·3	251·2	577·8	385·3	614·2	256·2	588·8	335·5	577·6	247·1	539·6	420·2
Fe (mg)	4·9	2·4	4·7	2·7	4·8	2·3	4·7	2·9	5·1	2·4	4·7	2·6
Zn (mg)	4·2	1·5	4·0	1·8	4·2	1·6	3·9	1·6	4·3	1·5	4·1	1·8
Thiamin (mg)	0·6	0·2	0·6	0·3	0·6	0·2	0·5	0·3	0·6	0·2	0·6	0·3
Riboflavin (mg)	0·9	0·4	0·9	0·6	0·9	0·4	0·9	0·5	0·9	0·4	0·9	0·6
Vitamin B_6_ (mg)	1·0	0·4	0·9	0·5	1·0	0·4	0·9	0·5	0·9	0·4	0·9	0·5
Vitamin C (mg)	58·8	34·4	51·2	35·8	56·9	29·0	49·9	28·7	60·7	38·9	51·7	38·2
Vitamin D (µg)	1·3	1·7	0·5	1·2	1·3	1·7	0·5	0·9	1·3	1·7	0·5	1·6
Vitamin E, α-TE (mg)	2·9	1·6	2·7	2·2	2·8	1·3	2·6	2·1	3·1	1·9	2·9	2·4
Retinol (µg)	128·2	91·3	105·0	129·5	135·2	100·7	108·8	128·8	121·6	81·5	102·1	125·5
RE (µg)	506·1	302·5	460·7	387·9	504·3	294·6	454·5	331·5	507·9	311·4	469·6	412·0
Niacin (mg)	6·9	3·2	6·2	4·1	6·7	3·3	5·9	3·7	7·2	3·2	6·2	4·3
Folate (µg)	116·6	48·4	106·4	60·3	115·9	50·4	102·6	63·9	117·3	46·7	109·2	55·0

IQR, interquartile range; α-TE, α-tocopherol equivalents; RE, retinol equivalents. Mean value was significantly different between sexes: *P<0·05.

The distribution of nutrient and energy intakes by percentile (5th, 25th, 75th and 95th), and minimum and maximum values, for all infants are reported in the online supplementary material (Tables S1, S2 and S3 for infants at 6, 9 and 12 months of age, respectively).

A wide range of variability in the intake values of some vitamins and minerals was observed in all infants at each follow-up. In particular, the mean daily intakes of Na and Ca were 373·1 (sd 321·5) mg and 383·5 (sd 184·0) mg at 6 months; 671·2 (sd 492·1) mg and 536·1 (sd 221·6) mg at 9 months; and 721·6 (sd 397·7) mg and 595·3 (sd 251·5) mg at 12 months, respectively.

The mean energy daily intake was greater in males than in females at all follow-ups, with a statistically significant difference at 12 months (3464·4 (sd 898·5) kJ *v*. 3242·8 (sd 907·6) kJ; *P*=0·04). Generally, the intakes of all twenty-eight nutrients considered were greater in males (Tables 2, 3 and 4), with the exception of Ca, that was always greater in females, without statistically significant differences, and thiamin, whose intake was very similar between sexes. A statistically significant difference between females and males was also observed in intakes of cholesterol (*P*=0·04) at 9 months and available carbohydrates (*P*=0·001), soluble carbohydrates (*P* = 0·01) and starch (*P*=0·03) at 12 months. No statistically significant difference was observed at 6 months.

The percentage contributions of the main macronutrients to total energy intake are reported in Table 5. In particular, available carbohydrates contributed ~50% of the total energy intake at each follow-up, while the contribution from total lipids ranged from ~40% at 6 months to ~34% at 12 months. The contribution of SFA to total energy intake ranged from 15·5% at 6 months to 13·7% at 12 months; the contribution of MUFA varied from 15·4% at 6 months to 12·4% at 12 months, while that of PUFA ranged from 5·1% at 6 months to 3·4% at 12 months.

**Table 5:** Percentage contribution of the main macronutrients to total energy intake at each follow-up among Italian infants in the Trieste Infants Food cohort

Nutrient	6 months	9 months	12 months	Italian DRV^(^ ^11^ ^)^	WHO (RDI)^(^ ^12^ ^)^
Total lipids (%)	40·2	36·0	33·8	40%E (AI)	30–40%E
SFA (%)	15·5	12·8	13·7	<10%E (STD)	–
MUFA (%)	15·4	13·5	12·4	for difference†	–
PUFA (%)	5·1	4·1	3·4	5–10%E (RI)	–
Available carbohydrates (%)	49·6	49·7	50·1	–	55–65%E
Soluble carbohydrates (%)	32·7	22·1	22·0	–	–
Starch (%)	7·7	15·0	18·1	–	–

DRV, Dietary Reference Value; RDI, Recommended Daily Intake; %E, percentage of energy intake; AI, Adequate Intake; STD, Suggested Dietary Target; RI, Reference Interval.

† The quantity of MUFA is calculated by difference between total lipids and the sum of SFA and PUFA.

**Fig. 2:**
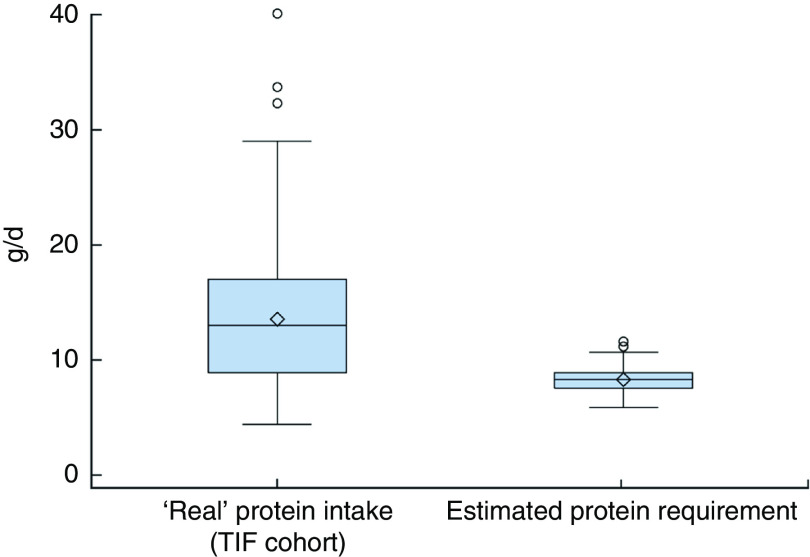
(colour online) Box-and-whisker plots comparing the ‘real’ protein intakes of Italian infants at 6 months of age in the Trieste Infants Food (TIF) cohort and their estimated protein requirements from the Italian Dietary Reference Values^(^ ^11^ ^)^ (calculated using the Average Requirement, equal to 1·11 g/kg weight per d, and the infants’ reported weights at 6 months). The bottom and top edge of the box represent the first and third quartiles (interquartile range); the line within the box represents the median; the open diamond represents the mean value; the ends of the bottom and top whiskers represent the minimum and maximum values; and the circles represent outliers

**Fig. 3:**
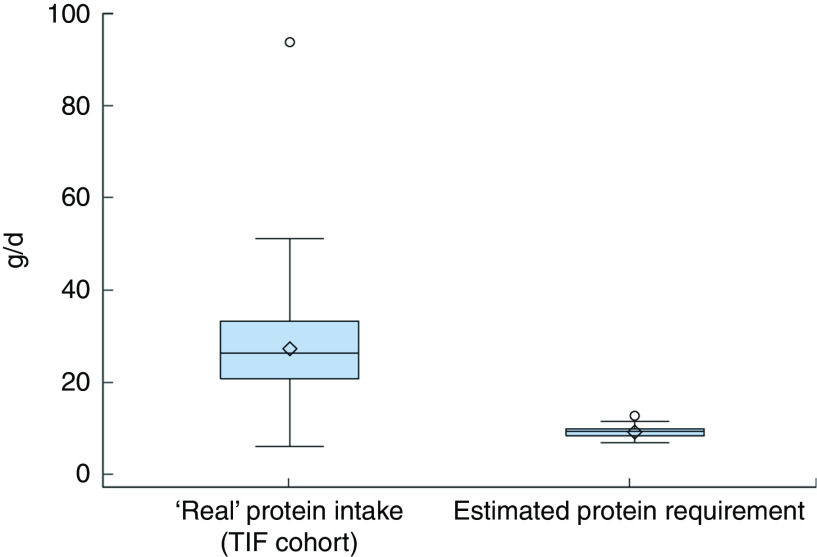
(colour online) Box-and-whisker plots comparing the ‘real’ protein intakes of Italian infants at 9 months of age in the Trieste Infants Food (TIF) cohort and their estimated protein requirements from the Italian Dietary Reference Values^(^ ^11^ ^)^ (calculated using the Average Requirement, equal to 1·11 g/kg weight per d, and the infants’ reported weights at 9 months). The bottom and top edge of the box represent the first and third quartiles (interquartile range); the line within the box represents the median; the open diamond represents the mean value; the ends of the bottom and top whiskers represent the minimum and maximum values; and the circles represent outliers

**Fig. 4:**
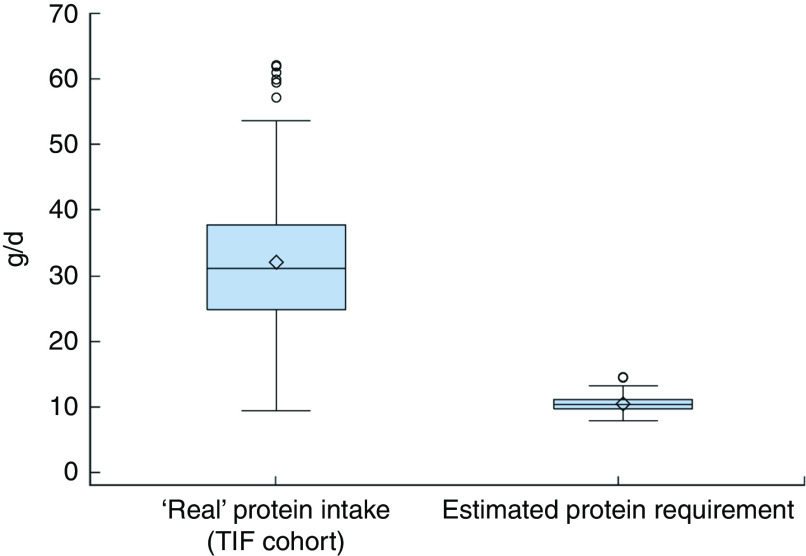
(colour online) Box-and-whisker plots comparing the ‘real’ protein intakes at of Italian infants 12 months of age in the Trieste Infants Food (TIF) cohort and their estimated protein requirements from the Italian Dietary Reference Values^(^ ^11^ ^)^ (calculated using the Average Requirement, equal to 1·11 g/kg weight per d, and the infants’ reported weights at 12 months). The bottom and top edge of the box represent the first and third quartiles (interquartile range); the line within the box represents the median; the open diamond represents the mean value; the ends of the bottom and top whiskers represent the minimum and maximum values; and the circles represent outliers

A comparison of the distribution of ‘real’ protein intakes in our cohort with the estimated protein requirements of our cohort from the Italian DRV^(^ ^11^ ^)^ at each follow-up is shown in Fig. 2, 3 and 4, respectively. The DRV are expressed as the Average Requirement, equal to 1·11 g/kg weight per d, and the estimated protein requirements were calculated using the infants’ weights reported in the questionnaires at 6, 9 and 12 months.

The distribution of protein intake, expressed as median and IQR, was greater in our cohort, when compared with estimated data using the Italian DRV^(^ ^11^ ^)^, at each follow-up: 13·1 (IQR 8·0) g/d *v*. 8·4 (IQR 1·3) g/d at 6 months; 26·4 (IQR 12·4) g/d *v*. 9·5 (IQR 1·5) g/d at 9 months; and 31·3 (IQR 12·7) g/d *v*. 10·8 (IQR 1·4) g/d at 12 months.

## Discussion

The present detailed nutritional evaluation, carried out in a cohort specifically recruited for this purpose, shows that energy and nutrient intakes were greater in male than in female infants, except for Ca and thiamin. This in line with reports from other studies^(^ ^8^ ^)^ but further analysis of food sources of nutrients may be needed to better explain these differences between sexes regarding dietary patterns and dietary choices in infants.

The contribution of total lipids to total energy intake (Table 5) appears to be in line with both DRV^(^ ^11^ ^)^ and WHO recommendations^(^ ^12^ ^)^, amounting to ~40% in infants aged 6–12 months. However, the energy contribution from PUFA appears to be lower than the DRV (5–10% of energy intake) at 9 and 12 months of age, while that of SFA appears to be higher than the DRV (<10% of energy intake) at all follow-ups. This could reflect an unbalanced diet with a low intake of essential fatty acids (*n*-3), in particular DHA and EPA that are important for neurodevelopment of infants. Regarding available carbohydrate intakes, values are lower than WHO recommendations^(^ ^12^ ^)^. Unfortunately, for this age range, no Italian reference data are available that can be used as a comparison for the analyses^(^ ^11^ ^)^. Moreover, the ‘real’ protein intakes of the TIF cohort (Figs 2, 3 and 4) were greater than the estimated protein requirements of the TIF cohort calculated using the Italian DRV^(^ ^11^ ^)^. The ‘real’ protein intakes cover the Average Requirement established using the Italian DRV but the values are two to three times greater. This could lead to short- and long-term negative health effects such as anticipation of the adiposity rebound, which normally occurs between 6 and 7 years of age, and an increased risk of kidney problems. The mineral consumption of the TIF cohort is very close to what is recommended, except for Na intake at 12 months (721·6 mg/d *v*. DRV of 400 mg/d), Fe intake at all follow-ups (from 3·5 mg/d at 6 months to 4·9 mg/d at 12 months *v*. DRV of 11 mg/d) and Ca intake at 6 months (383·5 mg/d *v*. WHO recommendation of 500 mg/d or DRV of 700 mg/d). Finally, the daily vitamin requirements are satisfied; the only exception is vitamin D whose daily intake decreases continually at the three follow-ups reaching a value that, at 12 months, is much lower than the reference value (1·3 mg/d *v*. DRV of 10 mg/d). The relative deficiency of Fe, Ca and vitamin D in the diet of infants in our cohort may be balanced by their higher bioavailability in breast milk and by exposure to sunshine as far as vitamin D is concerned^(^ ^18^ ^)^. This is another reason why it is important to prolong breast-feeding up to 2 years of life, as recommended^(^ ^4^ ^)^, and to include foods rich in Ca and Fe as first complementary foods.

To our knowledge, the present study is the first to provide a detailed description of the dietary intakes of energy, macro- and micronutrients in an Italian population of infants at different points during the first year of life and when complementary feeding is established. The main strengths of our study are the adoption of a rigorous design (prospective cohort study), which allowed the accuracy of data collection, the use of a 3d dietary record to collect dietary data and the extraction of food composition and nutritional data using Microdiet, a specifically dedicated software. Microdiet contains the Italian food composition database for epidemiological studies, that was compiled using standard methods established by the EuroFIR project (www.eurofir.org)^(^ ^13^ ^)^. These data were integrated with information from nutritional labels (e.g. baby food) and, in the case of human milk, from the literature^(^ ^14^ ^,^ ^15^ ^)^. This is an important tool for epidemiological research, public health nutrition and education, clinical practice and nutrition declaration of food labels.

The main study limitation was the loss to follow-up. However, no difference was seen in the main characteristics of women lost to follow-up compared with those who remained in the study, suggesting a random loss. Furthermore, the loss is comparable to that reported in other studies involving infants^(^ ^8^ ^)^. Moreover, the widespread use of baby foods, which are generally characterized by poor nutritional labelling, may have limited the precision with regard to the intakes of micronutrients such as vitamins and some minerals, while data concerning energy components (total proteins, available carbohydrates and total lipids) were completely covered^(^ ^17^ ^)^. The methodology adopted to estimate the consumption of breast milk (frequency of feeds, perceived length of each feed by mothers) and the use of a literature-derived composition constitute a weakness because it does not take into consideration inter- and intrasubject variability and may therefore contribute inaccuracy in the assessment of nutrient intakes^(^ ^17^ ^)^. Nevertheless, we decided not use the double weighing methodology (best method) because we think it is an invasive methodology and it goes against the main principle of breast-feeding. Our method has been used by other international infant cohort studies^(^ ^6^ ^,^ ^8^ ^)^. Finally, the nutritional comparison between our cohort’s nutrient intakes and the reference values^(^ ^11^ ^,^ ^12^ ^)^ may be hampered by the fact that the latter cover a wider age range (infants: 0·6–1 years). The mean daily energy and nutrient intake data reported for infants in the third Italian National Consumption Survey 2005–2006^(^ ^19^ ^)^ and in the National Health and Nutrition Examination Survey (NHANES) 2009–2012^(^ ^1^ ^)^ are comparable to the values we obtained for all infants, despite the fact that the two surveys cover wider age ranges: 0–2·9 years, and 6–11 months and 12–23 months, respectively. In 2014, an Italian group conducted a cross-sectional study (the Nutrintake 636 study) to compare the energy and nutrient intakes and anthropometric status of infants and toddlers living in North and South Italy, at different follow-ups^(^ ^20^ ^)^. Since they did not take into account the breast milk intake, we decided not to compare our results with theirs.

## Conclusions

The present descriptive paper provides detailed information on energy and nutrient intakes in an Italian population of infants during the first year of life. Understanding the dietary habits and the nutritional intake of infants during the first year of life is important because this period has a high impact on the development of food preferences that act on metabolic and chronic risk factors^(^ ^1^ ^,^ ^2^ ^)^. The results highlight a substantial adherence with Italian DRV and WHO recommendations^(^ ^11^ ^,^ ^12^ ^)^ concerning nutrient intakes, except the excessive protein intake, low vitamin D intake and the unbalanced intake of SFA and PUFA, that could lead to negative short- and long-term health consequences (e.g. obesity, rickets, kidney diseases, delays in neurodevelopment). However, further analyses will be carried out to identify dietary patterns and evaluate the adequacy of food choices in relation to national and international recommendations^(^ ^4^ ^,^ ^5^ ^,^ ^21^ ^)^.

## Supplementary material

For supplementary material accompanying this paper visit https://doi.org/10.1017/S136898001800201X. click here to view supplementary material
