# Supplementary material for: Nutrient intakes in an Italian population of infants during the complementary feeding period
Source: Public Health Nutr. 2018 Aug 30;21(16):3018–26. doi: 10.1017/S136898001800201X (PMC6190068; doi:10.1017/S136898001800201X)
Supplement: Supplementary file 1 [file S136898001800201Xsup001.doc]

**Table S1. Daily macro- and micronutrients and Energy intakes at 6 months** (n=268)

| **Nutrient** | **Minimun** | **Maximum** | **5 pctl** | **25 pctl** | **75 pctl** | **95 pctl** |
| --- | --- | --- | --- | --- | --- | --- |
| Energy (kJ) | 1105.4 | 6736.4 | 1309.0 | 1888.3 | 2775.6 | 3884.3 |
| Total proteins (g) | 4.5 | 108.1 | 6.1 | 10.0 | 19.2 | 32.4 |
| Total lipids (g) | 8.7 | 63.0 | 14.4 | 20.0 | 29.5 | 40.1 |
| Saturated fatty acids (g) | 0.5 | 19.6 | 5.8 | 7.6 | 11.0 | 15.0 |
| Monounsaturated fatty acids (g) | 0.34 | 25.8 | 4.8 | 7.4 | 11.8 | 17.5 |
| Oleic acid (g) | 0.3 | 26.4 | 4.7 | 7.2 | 11.2 | 16.9 |
| Polyunsaturated fatty acids (g) | 0.1 | 8.7 | 1.5 | 2.2 | 3.9 | 6.7 |
| Linoleic acid (g) | 0.4 | 7.7 | 1.3 | 1.8 | 3.2 | 5.8 |
| Linolenic acid (g) | 0.1 | 2.6 | 0.4 | 0.6 | 1.1 | 1.9 |
| Available carbohydrates (g) | 30.1 | 190.2 | 38.3 | 58.2 | 91.9 | 134.5 |
| Soluble carbohydrates (g) | 2.1 | 107.7 | 30.0 | 38.4 | 55.3 | 71.0 |
| Starch (g) | 0.0 | 69.8 | 0.0 | 1.3 | 19.4 | 39.2 |
| Fibre (g) | 0.0 | 24.2 | 0.0 | 1.2 | 6.0 | 10.6 |
| Cholesterol (mg) | 0.0 | 170.8 | 0.0 | 21.3 | 93.7 | 137.6 |
| Sodium (mg) | 40.4 | 2323.1 | 95.0 | 170.4 | 487.9 | 1031.4 |
| Potassium (mg) | 189.0 | 2795.5 | 325.8 | 485.7 | 884.8 | 1581.0 |
| Calcium (mg) | 129.6 | 1262.5 | 156.4 | 237.8 | 482.8 | 723.7 |
| Iron (mg) | 0.2 | 54.5 | 0.3 | 0.8 | 5.8 | 8.1 |
| Zinc (mg) | 1.4 | 8.5 | 1.8 | 2.6 | 4.1 | 6.2 |
| Vitamin B1 (mg) | 0.1 | 5.5 | 0.1 | 0.2 | 0.7 | 1.0 |
| Vitamin B2 (mg) | 0.2 | 5.7 | 0.3 | 0.4 | 0.9 | 1.4 |
| Vitamin B6 (mg) | 0.1 | 5.9 | 0.1 | 0.2 | 0.7 | 1.3 |
| Vitamin C (mg) | 17.2 | 175.6 | 23.4 | 37.8 | 82.1 | 127.0 |
| Vitamin D (µg) | 0.0 | 12.8 | 0.1 | 0.2 | 5.3 | 9.2 |
| Vitamin E α-TE (mg) | 0.0 | 8.3 | 0.0 | 0.3 | 1.9 | 4.0 |
| Retinol (µg) | 0.0 | 568.7 | 0.0 | 0.2 | 151.0 | 419.0 |
| Retinol Eq. (µg) | 0.0 | 2106.5 | 0.0 | 9.7 | 402.8 | 1009.6 |
| Niacin (mg) | 0.7 | 74.0 | 0.9 | 1.7 | 5.6 | 1.0 |
| Folate (µg) | 24.5 | 338.6 | 40.6 | 65.7 | 116.7 | 193.3 |

Pctl, Percentile

**Table S2. Daily macro- and micronutrients and Energy intakes at 9 months** (n=179)

| **Nutrient** | **Minimun** | **Maximum** | **5 pctl** | **25 pctl** | **75 pctl** | **95 pctl** |
| --- | --- | --- | --- | --- | --- | --- |
| Energy (kJ) | 1219.7 | 5987.5 | 1803.4 | 2624.9 | 3625.5 | 4803.8 |
| Total proteins (g) | 5.9 | 93.7 | 12.0 | 20.7 | 33.8 | 46.9 |
| Total lipids (g) | 12.5 | 61.0 | 16.0 | 23.6 | 36.7 | 48.3 |
| Saturated fatty acids (g) | 3.3 | 25.8 | 5.6 | 8.2 | 12.3 | 17.7 |
| Monounsaturated fatty acids (g) | 2.8 | 25.9 | 5.1 | 8.3 | 13.9 | 20.0 |
| Oleic acid (g) | 0.6 | 25.1 | 3.9 | 7.6 | 13.3 | 19.5 |
| Polyunsaturated fatty acids (g) | 0.8 | 10.9 | 1.6 | 2.4 | 4.3 | 6.6 |
| Linoleic acid (g) | 0.6 | 9.6 | 1.2 | 1.9 | 3.5 | 5.5 |
| Linolenic acid (g) | 0.1 | 1.7 | 0.2 | 0.3 | 0.6 | 0.9 |
| Available carbohydrates (g) | 40.0 | 194.4 | 52.4 | 82.8 | 119.0 | 159.8 |
| Soluble carbohydrates (g) | 6.0 | 99.1 | 19.9 | 33.2 | 52.2 | 71.9 |
| Starch (g) | 0.0 | 87.7 | 3.1 | 15.8 | 40.3 | 65.9 |
| Fibre (g) | 0.0 | 18.5 | 1.9 | 4.5 | 9.1 | 14.3 |
| Cholesterol (mg) | 0.3 | 240.6 | 11.7 | 35.6 | 92.8 | 155.3 |
| Sodium (mg) | 96.2 | 3414.0 | 180.3 | 368.2 | 867.7 | 1464.4 |
| Potassium (mg) | 253.2 | 3807.7 | 423.0 | 732.5 | 1350.8 | 2015.5 |
| Calcium (mg) | 94.9 | 1340.6 | 219.9 | 363.7 | 677.0 | 946.7 |
| Iron (mg) | 0.3 | 198.0 | 1.3 | 2.8 | 7.2 | 10.2 |
| Zinc (mg) | 0.2 | 12.1 | 1.0 | 2.2 | 4.6 | 6.8 |
| Vitamin B1 (mg) | 0.1 | 1.8 | 0.2 | 0.4 | 0.8 | 1.1 |
| Vitamin B2 (mg) | 0.2 | 2.3 | 0.3 | 0.5 | 1.0 | 1.4 |
| Vitamin B6 (mg) | 0.1 | 2.7 | 0.2 | 0.6 | 1.0 | 1.6 |
| Vitamin C (mg) | 18.1 | 189.3 | 27.1 | 45.3 | 94.1 | 132.7 |
| Vitamin D (µg) | 0.1 | 11.3 | 0.1 | 0.2 | 4.3 | 6.8 |
| Vitamin E α-TE (mg) | 0.0 | 7.5 | 0.4 | 1.6 | 3.7 | 6.0 |
| Retinol (µg) | 0.0 | 539.4 | 4.5 | 26.2 | 95.6 | 218.2 |
| Retinol Eq. (µg) | 0.0 | 1959.4 | 52.4 | 223.2 | 726.8 | 1311.2 |
| Niacin (mg) | 1.0 | 18.9 | 2.0 | 4.0 | 8.3 | 13.0 |
| Folate (µg) | 27.7 | 382.5 | 53.0 | 79.6 | 144.9 | 241.9 |

Pctl, Percentile

**Table S3. Daily macro- and micronutrients and Energy intakes at 12 months** (n=176)

| **Nutrient** | **Minimun** | **Maximum** | **5 pctl** | **25 pctl** | **75 pctl** | **95 pctl** |
| --- | --- | --- | --- | --- | --- | --- |
| Energy (kJ) | 1263.3 | 6514.4 | 2019.8 | 2818.4 | 3845.3 | 4884.4 |
| Total proteins (g) | 9.8 | 61.9 | 15.4 | 25.0 | 38.4 | 56.1 |
| Total lipids (g) | 10.4 | 72.9 | 15.1 | 23.8 | 35.4 | 49.6 |
| Saturated fatty acids (g) | 1.8 | 32.3 | 6.4 | 8.6 | 14.9 | 20.2 |
| Monounsaturated fatty acids (g) | 2.2 | 38.3 | 4.5 | 8. | 13.4 | 20.0 |
| Oleic acid (g) | 0.0 | 36.3 | 3.7 | 7.4 | 12.2 | 17.8 |
| Polyunsaturated fatty acids (g) | 0.5 | 9.6 | 1.3 | 2.0 | 3.7 | 5.9 |
| Linoleic acid (g) | 0.3 | 8.6 | 1.0 | 1.5 | 3.1 | 4.9 |
| Linolenic acid (g) | 0.1 | 1.1 | 0.2 | 0.3 | 0.6 | 0.9 |
| Available carbohydrates (g) | 38.6 | 205.8 | 59.2 | 85.2 | 130.0 | 159.9 |
| Soluble carbohydrates (g) | 0.3 | 92.1 | 22.5 | 37.6 | 54.5 | 76.9 |
| Starch (g) | 0.9 | 116.9 | 8.7 | 19.0 | 49.8 | 79.8 |
| Fibre (g) | 0.5 | 17.1 | 1.9 | 4.8 | 9.0 | 12.6 |
| Cholesterol (mg) | 0.0 | 363.8 | 23.0 | 63.3 | 119.8 | 223.0 |
| Sodium (mg) | 175.3 | 2148.2 | 257.7 | 426.5 | 894.0 | 1447.7 |
| Potassium (mg) | 128.6 | 2902.7 | 584.9 | 912.6 | 1616.0 | 2198.6 |
| Calcium (mg) | 148.0 | 1539.6 | 208.5 | 388.3 | 773.6 | 995.3 |
| Iron (mg) | 1.0 | 14.5 | 1.7 | 3.4 | 6.1 | 9.7 |
| Zinc (mg) | 1.2 | 10.1 | 2.2 | 3.1 | 4.9 | 7.6 |
| Vitamin B1 (mg) | 0.2 | 1.5 | 0.2 | 0.4 | 0.7 | 1.0 |
| Vitamin B2 (mg) | 0.3 | 2.0 | 0.4 | 0.6 | 1.2 | 1.5 |
| Vitamin B6 (mg) | 0.2 | 2.0 | 0.4 | 0.7 | 1.2 | 1.7 |
| Vitamin C (mg) | 11.0 | 237.1 | 18.9 | 36.2 | 71.9 | 121.3 |
| Vitamin D (µg) | 0.0 | 7.2 | 0.1 | 0.2 | 1.5 | 5.4 |
| Vitamin E α-TE (mg) | 0.0 | 11.5 | 0.8 | 1.7 | 3.9 | 5.8 |
| Retinol (µg) | 0.0 | 498.0 | 11.5 | 59.2 | 188.7 | 279.0 |
| Retinol Eq. (µg) | 26.4 | 1703.2 | 100.1 | 274.9 | 662.8 | 1079.3 |
| Niacin (mg) | 1.5 | 20.5 | 2.6 | 4.7 | 8.8 | 12.7 |
| Folate (µg) | 18.9 | 288.3 | 52.7 | 83.8 | 144.2 | 213.1 |

Pctl, Percentile
